# Spotlight on the Underdogs—An Analysis of Underrepresented *Alternaria* Mycotoxins Formed Depending on Varying Substrate, Time and Temperature Conditions

**DOI:** 10.3390/toxins8110344

**Published:** 2016-11-19

**Authors:** Theresa Zwickel, Sandra M. Kahl, Horst Klaffke, Michael Rychlik, Marina E. H. Müller

**Affiliations:** 1 Federal Institute for Risk Assessment (BfR), Max-Dohrn-Str 8-10, Berlin 10589, Germany; horst.klaffke@bfr.bund.de; 2 Leibniz-Centre for Agricultural Landscape Research (ZALF), Institute of Landscape Biogeochemistry, Eberswalder Str. 84, Müncheberg 15374, Germany; sakahl@uni-potsdam.de; 3 Technische Universität München, Chair of Analytical Food Chemistry, Alte Akademie 10, Freising 85354, Germany; michael.rychlik@tum.de; 4 University of Potsdam, Maulbeerallee 1, Potsdam 14469, Germany

**Keywords:** *Alternaria infectoria*, *A. tenuissima*, mycotoxin profile, wheat, rice, *Alternaria* toxin sulfates, modified *Alternaria* toxins, altertoxins, altenuic acid, HPLC-MS/MS

## Abstract

*Alternaria (A.)* is a genus of widespread fungi capable of producing numerous, possibly health-endangering *Alternaria* toxins (ATs), which are usually not the focus of attention. The formation of ATs depends on the species and complex interactions of various environmental factors and is not fully understood. In this study the influence of temperature (7 °C, 25 °C), substrate (rice, wheat kernels) and incubation time (4, 7, and 14 days) on the production of thirteen ATs and three sulfoconjugated ATs by three different *Alternaria* isolates from the species groups *A. tenuissima* and *A. infectoria* was determined. High-performance liquid chromatography coupled with tandem mass spectrometry was used for quantification. Under nearly all conditions, tenuazonic acid was the most extensively produced toxin. At 25 °C and with increasing incubation time all toxins were formed in high amounts by the two *A. tenuissima* strains on both substrates with comparable mycotoxin profiles. However, for some of the toxins, stagnation or a decrease in production was observed from day 7 to 14. As opposed to the *A. tenuissima* strains, the *A. infectoria* strain only produced low amounts of ATs, but high concentrations of stemphyltoxin III. The results provide an essential insight into the quantitative in vitro AT formation under different environmental conditions, potentially transferable to different field and storage conditions.

## 1. Introduction

The genus *Alternaria* contains pathogenic and saprophytic fungi with a considerable number of host plants such as ornamental and crop plants, fruits and vegetables [1,2]. Saprophytic *Alternaria* strains can be found on grains in storage included in postharvest spoilage processes or in the soil as microbiome members in the rhizosphere [3,4]. Pathogenic *Alternaria* species affect healthy plants and seeds inducing necrosis on leaves, reducing grain germination and can also cause stem cancer, leaf blight or leaf spot diseases [3,5,6].

During its growth the fungus may produce a vast number of mycotoxins, host-specific as well as non-host specific, which are associated with the infection and colonization of its plant substrates [7,8]. So far it is known that *Alternaria* strains are capable of producing over seventy secondary metabolites. However, the chemical structures of only a few of them have been elucidated. *Alternaria* toxins (ATs) differ widely in their chemical structures and therefore can be divided into five groups [9]:

- Dibenzo-α-pyrone derivatives: e.g., alternariol (AOH), alternariol mono methylether (AME), altenuene (ALT), isoaltenuene (isoALT) and altenuisol (ATL) whose originally proposed structure [10] has been recently revised [11];
- Tetramic acid derivatives: e.g., tenuazonic acid (TeA);
- Perylene quinone derivatives: e.g., altertoxin I, II (ATX-I, -II) and stemphyltoxin III (STTX-III);
- Aminopentol esters: host-specific ATs that are produced by the fungus *Alternaria alternata* f. sp. *lycopersici* and collectively known as the AAL toxins, e.g., AAL TB1 and TB2;
- Miscellaneous structures: e.g., tentoxin (TEN) a cyclic tripeptide and altenuic acid III (AA-III) a resorcylic acid substituted with butenolide and a second carboxylic acid in the side-chain [12,13].

Recently, so-called modified mycotoxins [14] such as sulfates and glucosides of AOH and AME (e.g., AOH-3-sulfate, AME-3-β,d-glucoside) were identified and synthesized [15,16].

Due to the ubiquitous occurrence of *Alternaria* these mycotoxins can be present as natural contaminants throughout the entire food and feed chain. Among the various produced mycotoxins AOH, AME, ALT, TeA and TEN are the best examined ones and can be frequently found in a broad spectrum of foodstuff commodities such as cereal products, vegetables, fruits and oil seeds [3,4,9,17,18].

The occurrence as food and feed contaminants is a cause of concern as some of the ATs are suspected to pose a serious health risk to humans and animals. The European Food Safety Authority (EFSA) classified AOH and AME as genotoxic substances. TeA, however, was classified as non-genotoxic, but due to its acute oral toxicity in mice and rats it was classified as possibly harmful. TEN is considered as non-harmful [9]. According to Frizzell et al. [19] AOH has the potential to modulate the human endocrine system by altering the hormone production and interfering with gene and receptor expression. Furthermore, AOH and AME inhibit the progesterone secretion of porcine ovarian cells in vitro, which may affect reproductive performance in livestock [20]. Studies focusing on the toxicity of other *Alternaria* mycotoxins remain scarce: Alternariol 5-*O*-sulfate demonstrated lower bioactivity than free AOH, but higher bioactivity compared to free AME in in vitro screening for cytotoxic activity [15]. Mutagenic effects of ATX-I-III and STTX-III were determined by in vitro assays [21,22]. ATX-II also demonstrated the highest toxicity against HeLa cells followed by AOH and ATL [23]. Furthermore, ATX-II has a 50-fold higher mutagenic potency compared to AOH and has been reported to induce gene mutations and DNA strand breaks in V79 cells, however it does not interfere with the cell cycle [24]. The genotoxic effects of ATX-II in human cells also far exceeded the effects of the main toxins AOH and AME [25]. A carcinogenic potential has been proven as well for the occurrence of *A. alternata* and its mycotoxins, AOH and AME, causing esophageal cancer [8,26,27]. However, ATX-II and STTX-III have only recently been considered to be more likely responsible for esophageal cancer than AOH and AME. The genotoxic potential of the perylene quinones with an epoxide group is stated to be probably caused by the formation of DNA adducts. The DNA strand breaks induced by ATX-II and STTX-III were more persistent than the ones induced by AOH [28].

The few in vitro studies on the influence of environmental conditions on the fungus growth and the mycotoxin production capability have been reviewed [4,17,29]. Toxins most frequently analyzed were AOH, AME and TeA, followed by ALT and ATX-II all of which are produced by *A. alternata* or *A. tenuissima.* The temperature and water activity (a_w_) level optima for these toxins were generally around 25 to 30 °C and 0.98 [4,17,29]. An Argentinean study showed that 75% of 87 *Alternaria* strains isolated from tomato, wheat, blueberry and walnut were able to produce mycotoxins. AOH and AME were the most common metabolites followed by TEN, ATX and TeA, and several qualitatively detected further metabolites [30]. The production of five different ATs by the same *Alternaria* isolates was compared in an in vitro assay, in cabbage, cultivated rocket *(Eruca sativa)* and cauliflower. AOH, AME, ATL and TEN showed a good correlation, while TeA was only produced in higher levels in liquid culture [31]. A difference in mycotoxin production between *Alternaria* species groups could be shown as well: strains characterized as *A. infectoria* isolated from wheat can be differentiated from other *Alternaria* species groups by low production of AOH, AME, ALT, ATX-I and TeA, by the color of cultivated colonies and by molecular classification [32].

The co-occurrence of several ATs is not only reported in in vitro studies but also in food and feed samples [9,17,18]. A few HPLC-MS/MS multi-methods have been published for the simultaneous quantification of the main ATs TeA, AOH, AME, ALT, TEN [33] and underrepresented ones such as ATX-I in tomato products [34], additionally ATX-II, STTX-III, ATL, isoALT, AA-III and AAL toxins TB1 and TB2 in fruit juices, vegetable juices and wine [13], in bakery products, sunflower seeds and vegetable oils [35], further including conjugated toxins of AOH and AME in cereal products [36] and also stable isotope dilution assay for several ATs in various commercial food samples [37].

To conclude, *Alternaria* species play not only an important role in plant pathology of agronomic crops with losses of harvest but also in food and feed quality and safety. For a reliable risk assessment of ATs in agricultural and processed food products it is also crucial to know more about the influencing factors during the mycotoxin producing processes.

**Figure 1:**
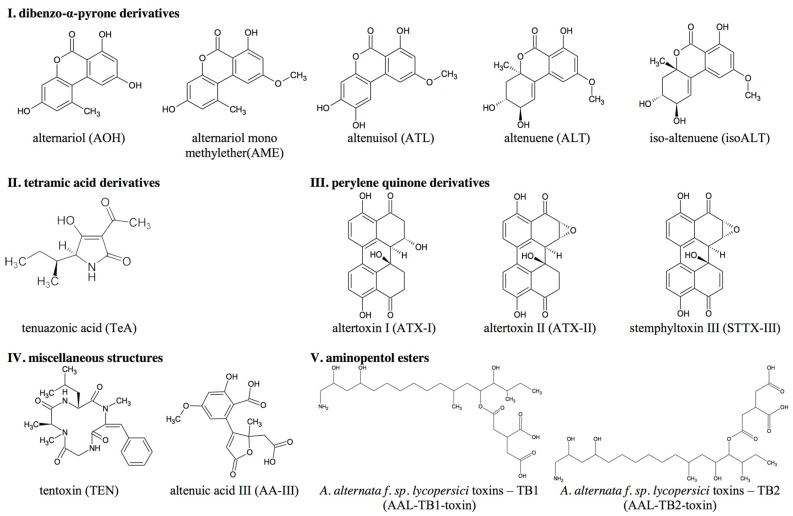
Chemical structures of *Alternaria* toxins (ATs) determined in this study.

The present study reports an overview of the quantitative production of AOH, AME, ALT, TeA, TEN, ATX-I and of the underrepresented toxins ATX-II, STTX-III, isoALT, ATL, AA-III and AAL TB1 and TB2 (Figure 1) under combinations of different growth conditions such as temperature, incubation time and nutrient media in in vitro cultures. Additionally, individual differences in mycotoxin production of three *Alternaria* strains from two species groups are examined as well. Furthermore, screening for modified ATs was carried out.

## 2. Results

The three *Alternaria* strains produced a highly variable mycotoxin cocktail on both substrates with up to eleven components in widely differing concentrations depending on the environmental conditions investigated in this study. Optimal conditions (rice as nutrient medium, 25 °C, 14 days of growth) trigger the production of a complex mixture of eleven or seven mycotoxins by both *A. tenuissima* strains or by the *A. infectoria* strain, respectively. Neither of the AAL-TB toxins were detected under either of the conditions. However, AOH-, AME- and ATL-sulfates could be tentatively identified at 25 °C in rice and in wheat. The composition of this cocktail and the amount of toxin produced was remarkably influenced by temperature, incubation time and the *Alternaria* species group and—to a lesser extent—by nutrient media used. In all cases, the underdogs within the ATs were analyzed as an essential part in these mixtures.

**Figure 2:**
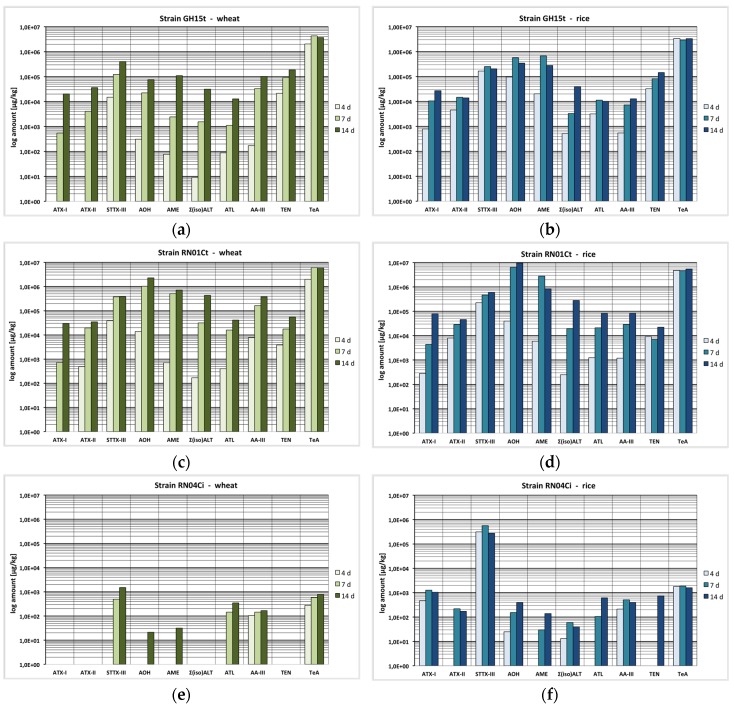
Production of altertoxin I (ATX-I), altertoxin II (ATX-II), stemphyltoxin III (STTX-III), alternariol (AOH), alternariol mono methylether (AME), sum of altenuene and isoaltenuene (Σ(iso)ALT), altenuisol (ATL), altenuic acid III (AA-III), tentoxin (TEN) and tenuazonic acid (TeA) after 4, 7 and 14 days at 25 °C; please note logarithmic scaling due to high difference in concentration between the toxins (μg·kg^−1^); (**a**) RN01Ct strain in wheat; (**b**) RN01Ct strain in rice; (**c**) GH15t strain in wheat; (**d**) GH15t strain in rice; (**e**) RN04Ci strain in wheat; (**f**) RN04Ci strain in rice.

**Figure 3:**
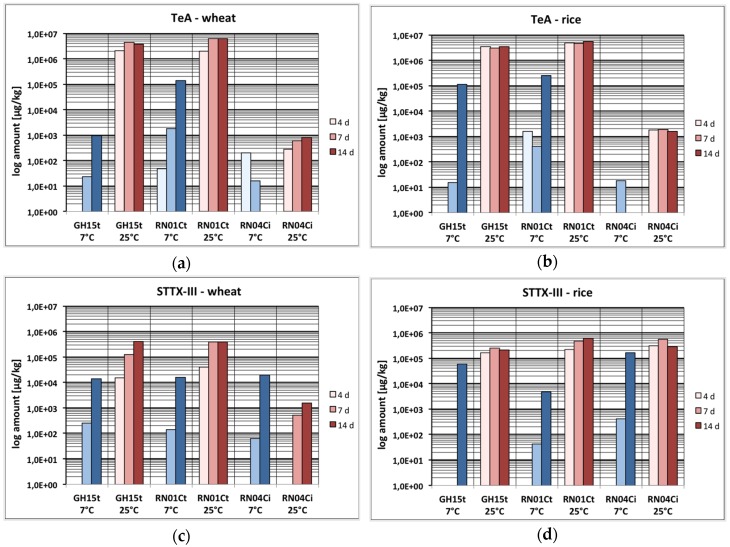
Production of tenuazonic acid (TeA) and stemphyltoxin III (STTX-III) at different temperatures after 4, 7 and 14 days by GH15t, RN01Ct and RN04Ci strains; please note logarithmic scaling due to high difference in concentration depending on the temperature (μg·kg^−1^); (**a**) Comparison of TeA production at 7 °C and 25 °C in wheat; (**b**) Comparison of TeA production at 7 °C and 25 °C in rice; (**c**) Comparison of STTX-III production at 7 °C and 25 °C in wheat; (**d**) Comparison of STTX-III production at 7 °C and 25 °C in rice.

**Figure 4:**
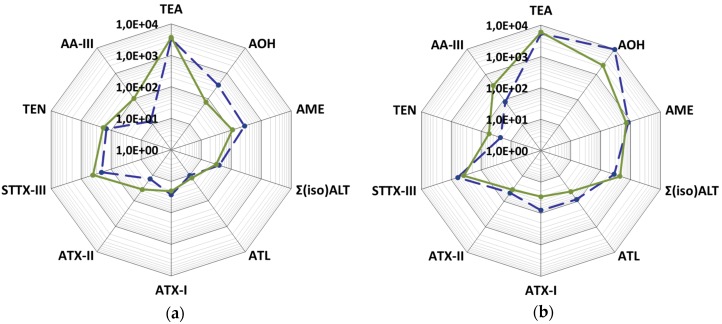
*Alternaria* toxin profiles of altertoxin I (ATX-I), altertoxin II (ATX-II), stemphyltoxin III (STTX-III), alternariol (AOH), alternariol mono methylether (AME), sum of altenuene and isoaltenuene (Σ(iso)ALT), altenuisol (ATL), altenuic acid III (AA-III), tentoxin (TEN) and tenuazonic acid (TeA) after 14 days at 25 °C in wheat and rice; please note logarithmic scaling of the contents (mg·kg^−1^); solid green lines: rice kernels; dashed blue lines: wheat kernels; (**a**) *Alternaria* toxin profile of GH15t ; (**b**) *Alternaria* toxin profile of RN01Ct.

**Figure 5:**
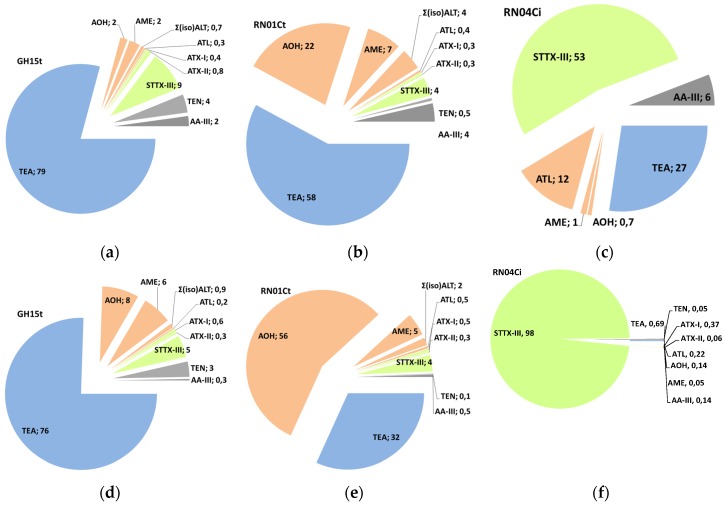
Distribution of *Alternaria* toxin production in percentage after 14 days at 25 °C in wheat kernels (**a**–**c**) and in rice kernels (**d**–**f**) by the *Alternaria tenuissima* strain GH15t (**a**,**d**), the *Alternaria tenuissima* strain RN01Ct (**b**,**e**) and the *Alternaria infectoria* strain RN04Ci (**c**,**f**).

Results are presented as mean values of triplicates with logarithmic scaling because of high differences in concentration between the toxins produced by the strains in Figure 2, Figure 3 and Figure 4 and are summarized as mean values in mg·kg^−1^ ± standard error of the mean in mg·kg^−1^ in Tables S1 and S2 available in the supplementary material. Percental distribution of the ATs depending on the strain are shown in Figure 5.

### 2.1. Mycotoxin Production Depending on the Incubation Time

To observe the effect of the incubation time on the toxin production, three different duration times (4, 7 and 14 days) were chosen to allow the fungus to grow and produce secondary metabolites (Figure 2a–f; Tables S1 and S2). In general, we observed a steady increase of AT concentrations from day 4 to day 14 with different rates depending on each secondary metabolite. Few exceptions could be observed with a decreasing phase between day 7 and day 14.

Most striking at 7 °C was the production of TeA by RN01Ct after 4 and 7 days in very low amounts in wheat and rice (0.046/1.8 and 1.6/0.41 mg·kg^−1^) followed by a considerable increase up to 139 and 252 mg·kg^−1^ after 14 days. The same was observed for STTX-III, which firstly could not be detected after 4 days, and after 7 days only in small amounts (0.40 mg·kg^−1^ in rice by RN04Ci) but appeared after 14 days with 163 mg·kg^−1^ (Table S1).

Contrasting the results under low temperature conditions, an increasing amount of all examined secondary metabolites was found at 25 °C with advancing incubation time. TEN production increased constantly from day 4 to day 7 by a factor of 4.5 from 21 to 92 mg·kg^−1^ and finally doubled from day 7 to 14 up to 185 mg·kg^−1^ in wheat and similarly in rice (GH15t). For AA-III, ATX-I and the sum of ALT and isoALT (Σ(iso)ALT), a steady increase in production by the strains GH15t and RN01Ct in wheat and rice was observed over the whole period as well. The same was determined for ATX-II, ATL and AOH, except in rice (GH15t), the production of ATX-II and ATL stagnated and the AOH concentration decreased by 40% from day 7 to 14. Likewise, AME was formed in increasing amounts in wheat by the GH15t and RN01Ct strains (0.078/2.4/110 mg·kg^−1^ and 0.71/506/717 mg·kg^−1^, respectively), however in rice a 60% (GH15t) or 70% (RN01Ct) decline in the total amount of AME was observed from day 7 to 14 (Figure 2a–d; Table S2).

In contrast to this, an increase of 100% and even 200% in TeA amount in wheat between day 4 and day 7 (2053 to 4309 mg·kg^−1^ GH15t; 1984 to 6098 mg·kg^−1^ RN01Ct) was detected while a 14% decline (3706 mg·kg^−1^; GH15t) or a stagnation (5995 mg·kg^−1^ RN01Ct) in the total amount of TeA was observed from day 7 to day 14. In rice, TeA was formed in similar amounts over the whole incubation time period (3358, 2946 and 3327 mg·kg^−1^; GH15t) or with a slight increase from day 7 to 14 (4637 to 5464 mg·kg^−1^; RN01Ct) (Figure 2a–d; Table S2).

The STTX-III concentration increased from day 4 to 7 by a factor of 2 or 10 in rice or wheat (225 to 470 mg·kg^−1^; 39 to 380 mg·kg^−1^) and by another 30% from day 7 to 14 in rice, but stagnated in wheat (RN01Ct) (Figure 2c,d). Strain GH15t produced STTX-III in increasing amounts over the whole incubation time in wheat but showed a decrease of 18% from day 7 to 14 in rice (251 to 206 mg·kg^−1^) (Figure 2a). The same can be stated for strain RN04Ci where a 50% decline in the total amount of STTX-III from day 7 to 14 in rice was determined (563 to 277 mg·kg^−1^) (Figure 2f; Table S2).

### 2.2. Mycotoxin Production Depending on the Temperature

The samples were incubated at two different temperatures: at 7 °C and at 25 °C. Overall, the toxin production was heavily influenced by this factor resulting in either formation of all eleven ATs in huge amounts up to g·kg^−1^ at 25 °C or a strongly delayed formation of only a few ATs (TeA, AA-III, AOH, STTX-III) in small quantities (μg·kg^−1^) at 7 °C. None of the other investigated *Alternaria* toxins could be detected at 7 °C in rice or wheat. This was the case after 4, 7 and 14 days. Only in one case did the toxin concentration achieve a similar or even higher level at 7 °C compared to 25 °C after 14 days (STTX-III in rice by strain RN04Ci) (Figure 3c,d).

AA-III, TeA and AOH were produced at 7 °C in low amounts after 4 days (0.011 mg·kg^−1^–1.58 mg·kg^−1^). After 7 days, in addition to these three toxins, STTX-III was found (0.041 mg·kg^−1^–0.24 mg·kg^−1^). After 14 days, the RN01Ct strain had produced TeA in wheat and rice up to 139 and 252 mg·kg^−1^ (Figure 3a,b), respectively, whereas the RN04Ci strain had produced STTX-III up to 163 mg·kg^−1^ in rice (Figure 3c,d). However, AA-III and AOH could still only be determined in small amounts below 0.2 mg·kg^−1^ (Table S1).

In comparison to this, all examined toxins were detected at 25 °C, most of them in small amounts already after 4 days. Generally, it can be stated that ATX-I, ATX-II and ATL were produced in comparably small quantities over the whole incubation time followed by AA-III, TEN, Σ(iso)ALT, STTX-III and AME in significantly higher amounts, whereas TeA and AOH were produced in large quantities, with maximum amounts of 6098 mg TeA kg^−1^ after 7 days in wheat (RN01Ct) and 9693 mg AOH kg^−1^ after 14 days in rice (RN01Ct), respectively. Both *A. tenuissima* strains showed similar toxin formation capability, whereas the *A. infectoria* strain showed little toxin production at 25 °C for the whole incubation period, except for STTX-III, which could be found in amounts up to 563 mg·kg^−1^ after 7 days in rice. All other toxins were only produced between 0.01 and 1.9 mg·kg^−1^ (Figure 2e,f; Table S2).

### 2.3. Mycotoxin Production Depending on the Substrate

To test the influence of different growth substrates, the three *Alternaria* strains were incubated in rice and wheat kernels. The substrate only marginally influenced the mycotoxin profile of the strains but the concentration of some toxins produced on the two different media varied up to ten-fold (Figure 4a,b).

For example, at 25 °C, AOH was produced by strain GH15t in higher concentrations in rice compared to in wheat after 4 (96 and 0.31 mg·kg^−1^), 7 (570 and 22 mg·kg^−1^) and 14 (346 and 74 mg·kg^−1^) days (Figure 2a,b and Figure 4a; Table S2). The same was observed for strain RN01Ct, which produced AOH in significantly higher concentrations in rice than in wheat after 4 (40 and 14 mg·kg^−1^), 7 (6452 and 1030 mg·kg^−1^) and 14 (9693 and 2289 mg·kg^−1^) days (Figure 2c,d and Figure 4b; Table S2). This trend was also determined for AME and ATX-I, but only with slightly higher amounts in rice for both *A. tenuissima* strains. In contrast to this, more AA-III could be detected in wheat than in rice after 4 (7.7 and 1.2 mg·kg^−1^), 7 (163 and 29 mg·kg^−1^) and 14 days (375 and 84 mg·kg^−1^) for strain RN01Ct, which was similar for strain GH15t. The production of the other toxins (ATX-II, STTX-III Σ(iso)ALT, ATL, TEN, TeA) varied in both substrates, mostly starting on day 4 with higher amounts in rice and then switching to a comparable or slightly higher toxin production in wheat after 7 or 14 days. For example TEN was produced in rice and wheat after 4 (9.1 and 3.9 mg·kg^−1^), 7 (7.1 and 18 mg·kg^−1^) and 14 days (22 and 55 mg·kg^−1^) by strain RN01Ct (Figure 2c,d and Figure 4b; Table S2). The toxin production capability of the *A. infectoria* strain RN04Ci was higher in rice than wheat both in concentrations and a number of ATs. In particular, STTX-III was produced in high concentrations in rice but not in wheat (Figure 2e,f; Table S2).

However, the mycotoxin profiles of the *A. tenuissima* strains GH15t and RN01Ct at 25 °C and after 14 days in rice reflect their profiles in wheat (Figure 4a,b).

### 2.4. Mycotoxin Production Dependent on the Strain

Two strains from the *A. tenuissima* species group (GH15t and RN01Ct) and one strain from the *A. infectoria* species group (RN04Ci) were chosen for the study. The two *A. tenuissima* strains were able to produce all eleven ATs and some of them in high amounts at 25 °C: 9693 mg·kg^−1^ AOH in rice substrate after 14 days, 6098 mg·kg^−1^ TeA in wheat kernels after 7 days and 470 mg·kg^−1^ STTX-III in rice after 7 days (Figure 5a,b,d,e). In comparison, the *A. infectoria* strain could form all examined ATs, but only in low concentrations under the same conditions: 0.4 mg kg^−1^ AOH and 0.6 mg kg^−1^ TeA, but remarkably, 563 mg·kg^−1^ of STTX-III (Figure 5c,f).

 The mycotoxin cocktails of the two *A*. species groups analyzed after 14 days at 25 °C showed remarkable differences: The main component of the cocktail produced by both *A. tenuissima* strains in wheat was TeA with 58% for strain RN01Ct followed by AOH (22%) and in descending order AME (7%), Σ(iso)ALT (4%), STTX-III (4%) and AA-III (4%) (Figure 5b). With regard to strain GH15t, the TeA fraction was even 79% followed by STTX-III (9%) and TEN (4%), AOH (2%), AME (2%) and AA-III (2%) (Figure 5a). Strain GH15t showed a similar toxin distribution in rice, whereas strain RN01Ct produced mostly AOH (56%) instead of TeA (32%) (Figure 5d,e).

In contrast, *A. infectoria* produced predominantly STTX-III (53%), followed by TeA (27%), ATL (12%), AA-III (6%), AME (1%) and AOH, all in low amounts up to 1.5 mg·kg^−1^ in wheat whereas in rice STTX-III was produced in higher amounts (277 mg·kg^−1^) almost exclusively (Figure 5c,f).

### 2.5. Modified Toxins

Due to the lack of commercially available analytical calibration standards of modified *Alternaria* toxins several experiments were carried out for identification (see Section 5.7). These experiments included precursor ion scans, neutral loss scans and collision induced mass spectra (MS2) in high-resolution or tandem mass spectrometry. AOH-, AME- and ATL-sulfates could be tentatively detected in extracts from rice and wheat, which were inoculated with strain GH15t or RN01Ct at 25 °C. Sulfates of ALT, isoALT, ATX-I, ATX-II or STTX-III or conjugations with glucoside could not be detected with the applied tandem mass spectrometry or high-resolution MS methods. Multiple reaction monitoring (MRM) transitions were monitored and the ion ratios of the selected ion transitions displayed no significant variances (<5%) within all positive samples. The earlier retention times of the substances in comparison to the retention times of the respective ATs is a further indication for the more water-soluble modified ATs (Table S3). The retention times of all quantified ATs were within a permitted tolerance of ±0.2 min in solvent calibration samples compared to matrix samples [13] and retention times of the sulfates were within this permitted tolerance in all positive samples.

Neutral loss scanning on a triple quadrupole mass spectrometer for semi-targeted detection of sulfoconjugated metabolites has been described before as a powerful screening tool, because many sulfated compounds show a characteristic neutral loss of 80 Da (SO_3_) [38].

The measured accurate masses of the sulfates were obtained with an accuracy of ±5 ppm compared to the calculated exact masses [16] (Table S4).

The appearance of AOH-sulfate was detected in rice after 7 days (strain GH15t). Strain RN01Ct appeared to produce AOH-sulfate over the whole incubation period in rice. The response of the signal increased by a factor of 6.5 from day 4 to day 7 and decreased by 65% from day 7 to day 14. On the contrary, the amount of AOH increased enormously (40/6452/9694 mg·kg^−1^) over the whole period. Strain RN01Ct produced AOH-sulfate on day 7 and day 14 in wheat, too. The response of the signal decreased by 70%; conversely, the AOH concentration rose by a factor of 2 (Table S2).

AME-sulfate was formed by strain GH15t after 14 days in wheat and between 7 and 14 days in rice. The response of the AME-sulfate signal decreased by 80% from day 7 to 14 as well as the amount of AME (674 to 278 mg·kg^−1^). Strain RN01Ct produced AME-sulfate after 4, 7 and 14 days in rice. At first the signal increased by a factor of 1.5, but showed a decline by 45% from days 7 to 14. Likewise, the AME concentration increased (6.0–2782 mg·kg^−1^) and decreased by 70% after 14 days (831 mg·kg^−1^). In wheat the AME-sulfate signal increased by a factor of 2.3 as the AME amount increased by a factor of 1.4 (506 to 717 mg·kg^−1^) from day 7 to 14 (Table S2).

ATL-sulfate was found only after 14 days and only in rice (RN01Ct).

## 3. Discussion

Our results show that different *Alternaria* sp. isolates from wheat are able to produce up to eleven *Alternaria* toxins under varying in vitro conditions in wheat and rice substrate. TeA is the most extensively produced toxin under nearly all conditions. The host-specific AAL toxins TB1 and TB2 were not detected at all as assumed. The obtained results of the study are in line with results obtained in previous in vitro studies relating to the major examined ATs TeA, AOH, AME, ALT and TEN [17,30,31]. TeA has been indicated as a major mycotoxin in naturally infected tomatoes, wheat and cereal-based foodstuffs [9,18]. *A. alternata* and *A. tenuissima* are regarded as highly potential toxin producers: 100% of strains isolated in the Mediterranean regions produced TeA, 93% AOH and AME [39]; all strains isolated in China from weather-damaged wheat were able to accumulate AOH and AME [40] as were all isolates from Poland [41], Italy [42], Argentina [43] and Germany [32]. The concentrations of AOH, AME and TeA analyzed in different in vitro experiments are in line with those reported in our study.

The production of mycotoxins is dependent on the growth conditions of the fungus: humidity (water activity), temperature, composition of nutrient media and pH conditions. Lee et al. [17] revised the optimum conditions of some abiotic factors for the growth and mycotoxin production of several *Alternaria* strains isolated from different host plants. Fungal development and AOH, AME and TeA formation were found within a wide temperature range (5–35 °C), water activity range (0.88–0.999) and under different pH conditions [44,45,46] independently of the host plants. Our results show the production of not only TeA but also STTX-III, AOH and AA-III at 7 °C and indicate the high likelihood of accumulating these toxins at cooler temperatures during the vegetation period of different vegetables, fruits and crop plants and under storage conditions. This may impair the capability to store agricultural commodities such as wheat kernels and possibly leads to a misjudgement of mycotoxin contamination. The present study not only summarizes the significance of the major toxins AOH, AME and TeA, but also highlights the formation of a multicomponent mycotoxin mixture under various abiotic conditions. Shier et al. [47] and Vesonder et al. [48] already described the risk of additional or synergistic effects caused by the co-occurrence of different mycotoxins. The simultaneous exposure to diverse *Alternaria* toxins should be linked to a greater and stronger adverse impact on human and animal health than indicated by a single mycotoxin. Therefore, the knowledge of the mycotoxin cocktail production influenced by environmental conditions is important in developing forecasting risk models in natural habitats.

During the present study, temperature, nutrient media and incubation time have influenced the production of all mycotoxins evaluated depending on the strain and its belonging to a species group of *Alternaria*. The obtained data on mycotoxin production in rice and wheat substrate verify the observations made by Kahl et al. which revealed the segregation of twenty-nine *A. infectoria* strains from *A. alternata*, *A. tenuissima* and *A. arborescens* due to the non-production of some toxins or to a different toxigenic pattern [32]. The entirely different mycotoxin profile of *A. infectoria* detected in the present study facilitates the singular position of this species group within the genus *Alternaria*. Furthermore, stemphyltoxin III is the only mycotoxin whose concentration is comparable in the *A. infectoria* as well as in the two *A. tenuissima* cultures. But the large share of STTX-III in the mycotoxin mixture of *A. infectoria* could be an explicit attribute for this species-group and may differentiate between the species groups. Moreover, STTX-III is a widely neglected metabolite among the *Alternaria* toxins though it has mutagenic potential [21]. However, the instability of STTX-III in solvent solution has been shown in a previous stability study by Zwickel et al. [13]. Thus similar analyses dealing with the production of STTX-III in *Alternaria* species are not known.

The formation of the modified mycotoxin—AOH-sulfate—showed no unequivocal pattern with regard to the formation of AOH. Between day 4 and day 7, the more AOH that was produced the more sulfate was detected, which was demonstrated by a higher response of the signals. However, from day 7 to day 14 the AOH-sulfate signal diminished even though the AOH concentration rose further. By comparison, AME-sulfate formation showed an analogy compared to the AME formation. After an increase in AME production, an increase in the respective sulfate was also observed. Likewise, a decline in the AME amount correlated with a decrease in the response of the AME-sulfate signal. ATL-sulfate was only detected in one sample after 14 days at 25 °C. ATL itself was generally produced in low amounts but in this sample it was produced in higher amounts. Therefore, the conclusion can be drawn that the sulfoconjugation is triggered depending on the concentration of the respective toxin. AOH- and AME-sulfates were previously reported in *Alternaria* sp. isolates from *Polygonum senegalense* grown either in liquid culture or in solid rice [15]. Recently, the formation of these two and an additional six new sulfoconjugated metabolites in cultures of *Alternaria* sp. was described by Kelman et al. [49]. The *Alternaria* isolates hereby were inoculated in liquid culture as well as rice and Cheerios (American brand of breakfast cereals). For identification high-resolution neutral loss filtering was used as well in this study.

In our study the rice and wheat kernels were sterilized twice by autoclaving at 121 °C. This procedure destroyed all metabolic activity of the substrate. Therefore, the enzymatic activity for phase-I and phase-II reactions could only be derived by the fungus itself. Therefore, the conjugation of the toxins AOH, AME and ATL with sulfate to more water-soluble molecules is a fungal reaction during the later stages of growth. One reason for this might be that the self-detoxification of the fungus as AOH and AME may also affect the fungal development. One hypothesis regarding the question as to why secondary metabolites including mycotoxins are formed by fungi is that these substances have extrinsic functions and may give the producers a competitive advantage towards other microorganisms and during the infection processes of the host cells [44,50,51]. Therefore, we hypothesize that the formation of sulfate derivatives inactivates or decreases the toxicity of the basic substances and promotes the faster excretion of the more water-soluble conjugates as a self-detoxification process.

In vitro studies should provide an indication of the behavior of fungal strains in a natural complex environment. Abiotic driving factors recognized in laboratory experiments influence the fungal growth and mycotoxin production in natural habitats too but in a more complex and multifactorial situation. *Alternaria* strains are members of a microbial community and a biotic network in the phyllosphere (aerial plant parts) and rhizosphere of different crop plants. Interactions of fungi and bacteria with *Alternaria* could exist more often in competition and in antagonistic processes than in undisturbed coexistence. Few studies dealing with the co-occurrence of *Fusarium* and fluorescent pseudomonads with *Alternaria* suggest the implication of mycotoxins as antagonistic substances in these interactions [52,53] Simultaneously, competition processes between different fungal genera affect the production of mycotoxins or increase the metabolization of toxins [54,55]. That means that the production of mycotoxins in natural habitats is influenced by biotic and abiotic factors and could represent various toxin profiles and different concentrations of mycotoxins [56,57].

In this cooperation work we conducted a further experiment with strains isolated from different habitats [32]. Also, the production of STTX-III in remarkably high amounts by *A. infectoria* strain will be monitored by investigation of several other isolated *A. infectoria* strains. The results of this next experiment will soon be published.

## 4. Conclusions

In general, fungi of the *Alternaria* species-group *tenuissima* and *infectoria* are able to produce a complex multicomponent mycotoxin cocktail. Temperature, nutrient media, incubation time and the fungal strain had a strong influence on what toxins were formed, the percentage they made up of the total mixture and the concentrations they were produced in. Here, we present the first in vitro study on the formation of underrepresented *Alternaria* toxins such as STTX-III, ATX-II, ATL, AA-III, AOH-sulfate, AME-sulfate and ATL-sulfate along with the better investigated ATs AOH, AME, ALT, TeA and TEN, depending on varying substrate, time and temperature conditions. It can thus be concluded that these underrepresented ATs could also be harmful substances in naturally contaminated crops, fruits and vegetables. A monitoring assessment of food and feeds should include the analyses of these underdogs. To our best knowledge this is the first time that the occurrence of sulfoconjugated altenuisol has been described.

## 5. Materials and Methods

### 5.1. Reagents, Solvents and Equipment

Analytical solid calibrants of AOH, AME, TEN, TeA and a mixture of AAL-TB1 and TB2 toxin, all >99% purity were purchased from Sigma-Aldrich, St. Louis, MO, USA. ATX-I, ATX-II and STTX-III were provided by the group of Professor Metzler at the Institute of Applied Bioscience (Karlsruher Institut für Technologie (KIT), Karlsruhe, Germany) via preparative HPLC of *Alternaria* isolates [58]. Crystalline ALT, iso(ALT), AA-III, and ATL were synthesized and provided by the group of Professor Podlech at the Institute of Organic Chemistry (KIT) [11,12]. Analytical reagent quality ammonium acetate, ammonium hydroxide solution (25%), LiChrosolv LC-MS hypergrade quality methanol and acetonitrile were purchased from Merck, Darmstadt, Germany. Analytical grade water (0.055 μS·cm^−1^) was generated from a Milli-Q system (Merck, Darmstadt, Germany).

### 5.2. Preparation of Standard Solutions

Stock solutions were prepared by dissolving 1 mg of the solid material in 1 mL methanol to obtain 1 mg·mL^−1^ stock solutions. Dried down standards (ATX-I, ATX-II and STTX-III) with a proposed absolute amount of 100 μg of the toxin were redissolved in 1 mL methanol. All stock solutions were kept at −30 °C. The actual concentrations were determined by ultraviolet–visible spectroscopy (UV/Vis) using a UV-1700 Pharma Spec (Shimadzu, Kyoto, Japan). Used wavelength and extinction coefficients for each toxin are described in a published work [13]. Mixed stock solutions with a concentration of 1 μg·mL^−1^ of each toxin were prepared beforehand in a mixture of 75% 1 mM ammonium acetate (pH 9) and 25% methanol. Calibration mix solutions were always prepared freshly in a range from 1 ng·mL^−1^ to 1000 ng·mL^−1^.

### 5.3. Instrumentation and Equipment

Analyses were performed using a 1100 HPLC system from Agilent Technologies (Santa Clara, CA, USA) coupled to an API 4000 (SCIEX, Foster City, CA, USA) triple quadrupole mass spectrometer. The system was equipped with an electrospray (ESI) interface (Turbo V^TM^, SCIEX, Foster City, CA, USA) and negative and positive ionization was used during acquisition. Chromatographic separation was performed on a 5 μm particle size, 100 mm × 2 mm i.d Gemini NX-C18 HPLC column from Phenomenex (Aschaffenburg, Germany). Data acquisition and evaluation were performed with Analyst version 1.6.2 (SCIEX, Foster City, CA, USA, 2013).

High-resolution mass spectrometry (HRMS) analyses to confirm the modified ATs (AOH-, AME- and ATL-sulfate) were performed on an Accela HPLC system coupled to an Exactive^TM^ (orbitrap) HCD (higher energy collisional dissociation) system fitted with a HESI II (heated-electrospray ionization) source (Thermo Fisher Scientific Inc., Waltham, MA, USA).

### 5.4. Fungal Isolates Used in this Study

*Alternaria*-infected grain samples were collected from winter wheat fields in the northeast Germany (Uckermark region) and central Russia (Novosibirsk region) in 2012. The fields underlie common agricultural practices and German sites take part in long-term examinations by the Leibniz-Centre for Agricultural Landscape Research Müncheberg, Germany (ZALF). *Alternaria* was isolated from grains after incubation on nutrient agar (potato dextrose agar, PDA, Carl Roth, Karlsruhe, Germany), morphologically identified and classified as belonging to the *A. infectoria* or *A. tenuissima* species group [32]. Single spore stock cultures of the isolates are maintained in sterile wheat mixtures at −20 °C. Three *Alternaria* isolates were chosen for the present mycotoxin analysis on the basis of previous mycotoxin measurements of ALT, AOH, AME, ATX-I and TeA by means of HPLC with diode-array detection (HPLC-DAD) analysis. A high (GH15t) and a medium *Alternaria* mycotoxin producer (RN01t) of the *A. tenuissima* species group were chosen additionally to one *A. infectoria* strain (RN04Ci), which could only produce low amounts of ATs [32].

### 5.5. Growth Conditions for Fungal Isolates

A spore suspension with 1 × 10^5^ spores mL^−1^ was produced for each isolate after incubation on potato-carrot-agar described by Kahl et al. [32]. A total of 114 centrifuge tubes (15 mL) were filled with 0.2 g of either rice (*n* = 57) or wheat kernels (*n* = 57) and 250 μL of demineralized water. Tubes were sterilized twice before inoculation. Three wheat and three rice samples as controls were not inoculated but incubated at 25 °C for 14 days to prove that there was no contamination. For each of the three *Alternaria* isolates 36 tubes—half of them containing wheat kernels the other half rice kernels—were inoculated with 50 μL of the respective spore suspension. Nine samples for each substrate group were incubated at 7 °C to simulate standard storage conditions and the other nine samples at 25 °C to simulate the conditions during the ripening process of cereals. Three samples of each temperature were incubated in darkness for 4, 7 and 14 days, respectively. After the respective incubation time samples were frozen at −20 °C to stop the growth of the fungus and any further mycotoxin production.

### 5.6. Extraction of Alternaria Toxins from Fungal Cultures

After defrosting, 5 mL of an acetonitrile/water/acetic acid mixture (79:20:1; *v*/*v*/*v*) were added to each sample tube. The fungus-kernel complex was mechanically pounded with a spatula. Ultrasonic extraction was carried out for 15 min. Subsequent tubes were roughly shaken at 25 °C for 60 min. The two steps were repeated one more time and after that the sample tubes were centrifuged at 4000 rpm for 6 min. Then, 2 mL of the supernatant were filled in 2 mL glass vials and stored at −30 °C until further processing.

### 5.7. HPLC-MS/MS Analysis of Alternaria Toxins

The ATs were simultaneously separated on a Gemini NX-C18 (2.1 × 100 mm, 5 μm) HPLC column equipped with a C18 SecurityGuard^TM^ cartridge system (0.3 mm) using binary linear gradient elution. Column oven temperature was set to 40 °C. Solvent A contained 1 mM of ammonium acetate and a pH adjusted to 9 with ammonium hydroxide solution (25%), while solvent B was methanol. The flow rate was set to 0.3 mL·min^−1^. Solvent B was 0% at 0 min, 0% at 1.0 min, 95% at 1.2 min, 95% at 6.0 min and 0% at 7.0 min. Equilibration time was set to 5 min with 100% of solvent A. Total run time was 12 min. The autosampler was operated at 10 °C and the injection volume was set to 5 μL. 2 mM ammonium acetate in methanol was used as post column solvent with a constant flow rate of 0.2 mL·min^−1^. The HPLC method has been precisely published by Zwickel et al. [13].

The mass spectrometer was operated in the negative electrospray ionization mode using multiple reaction monitoring (MRM) mode. Two ion transitions were scanned for each target compound. After the last analyte was detected, the polarity was switched to positive mode for 3 min to prevent the MS from possible contamination with negatively loaded matrix compounds [13]. The selected ion transitions with the optimized collision energies (CE), collision cell exit potential (CXP) and declustering potential (DP) for each analyte [13] are summarized in Table S3, entrance potential (EP) was set to −10 V and the gas for the collisionally activated dissociation (CAD) to 6 (arbitrary unit). Both quadrupoles (Q1 and Q3) were set to unit resolution. The source parameters were set as follows: curtain gas, 40 psi, ion spray voltage, −3500 V (0–7 min); +3500 (7.01–10 min); temperature 550 °C; spray gas (GS1), 35 psi; dry gas (GS2) 50 psi. Ultra high purity nitrogen (99.999%) was used as gas.

### 5.8. Neutral Loss Scan and HRMS Analysis of Modified Alternaria Toxins

Sulfoconjugates were detected in negative mode by monitoring constant losses of 80 Da (SO_3_) and precursors of *m*/*z* 80. Subsequently product ion scans of the detected precursors were obtained by collision-induced dissociation (CID) experiments. For each detected AT-sulfate two ion transition were selected—AOH-sulfate (*m*/*z* 337.1–*m*/*z* 257; *m*/*z* 337.1–*m*/*z* 213); AME-sulfate (*m*/*z* 351.1–*m*/*z* 271; *m*/*z* 351.1–*m*/*z* 256) and ATL-sulfate (*m*/*z* 353.1–*m*/*z* 273; *m*/*z* 353.1–*m*/*z* 230) (Table S3)—and integrated into the original method [13]. AOH- and AME-sulfate ion transitions correspond with the obtained transitions from analytical standard substances [36]. Ion ratios and retention times of detected sulfates in samples were monitored.

The detected precursor ions of AOH-, AME-sulfate were confirmed via high-resolution MS by comparing their calculated exact masses with the actual detected masses and the presented masses from the synthesized molecules [16] (Table S4). HESI parameters were set as follows: sheath gas flow 20 psi; spray voltage 4 kV, capillary temperature 350 °C; capillary voltage −60 V, tube lens voltage −120 V; skimmer voltage −25 V; heater temperature 350 °C. Ultra high purity nitrogen (99.999%) was used as gas. HRMS scan parameters were set as follows: scan range 150.00–1000.00 *m*/*z*; resolution was set to ultra-high; polarity was negative; AGC target was balanced.

### 5.9. Validation Parameters and Sample Preparation for HPLC-MS/MS Measurement

Limits of detection (LODs) and limits of quantification (LOQs) were determined according to DIN EN standard 32645 in extracts from not inoculated but incubated samples for all *Alternaria* toxins [13]. LODs ranged from 0.5 to 3.0 μg·kg^−1^ and LOQ ranged from 1.8–9.5 μg·kg^−1^ (see table S5).

External calibration curves were prepared in injection solution (1 mM ammonium acetate (pH 9): methanol; 75:25; *v*/*v*). Linear calibration curves ranged between 1.0–1000 ng·mL^−1^ for all analytes because of high differences in concentration between the toxins in the samples. Due to inhomogeneity of variance, what is almost inevitable for LC-ESI-MS/MS measurement when using a large calibration range is that the accuracy in the lower end of the range is not confidently given. Next to using weighted least squares linear regression (1/x) another alternative to counteract inhomogeneity of variance is to stagger calibration solutions and up to 20 samples and calculate the concentrations of the toxins with the preceding calculation curve. Both methods were compared and led to similar results.

Samples had to be diluted 1:2000 (14 day samples at 25 °C), 1:1000 (7 and 14 day samples at 25 °C), 1:500 and at least 1:100 (all other samples) with injection solution (1 mM ammonium acetate (pH 9): methanol; 75:25; *v*/*v*) due to high differences in concentration between the ATs. Every dilution approach was tested for matrix effects (ME), which may lead to ion suppression or ion enhancement. Therefore, the blank samples (not inoculated) were diluted equal to the inoculated samples and spiked with AT mix solution in three concentrations (1, 200 and 1000 ng·mL^−1^). The effect of the presence of matrix on each analyte was calculated by dividing the analyte peak areas of matrix-matched standard samples through the peak areas of standard samples in pure injection solution: ME (%) = peak area of analyte in matrix solution/peak area of analyte in neat solution – 1) × 100 [13]. A negative ME indicates ion suppression and a positive one ion enhancement. For each analyte in each dilution approach a ME < ±10% was calculated. Additionally, samples with high toxin contents were diluted 2000, 1000, 500 and 100 times. Calculation via external calibration led to comparable results. Hence, the dilution of the samples resulted in a sufficient dilution of the matrix and no further compensation measures were needed.

## Supplementary Materials

The following are available online at www.mdpi.com/2072-6651/8/11/344/s1, Table S1: Mean content (*n* = 3) of produced *Alternaria* toxins at 7 °C after 4, 7 and 14 days in wheat and in rice in mg kg^−1^ (± standard error of the mean; n.d. not detected), Table S2: Mean content (*n* = 3) of produced *Alternaria* toxins at 25 °C after 4, 7 and 14 days in wheat and in rice in mg kg^−1^ (± standard error of the mean; n.d. not detected), Table S3: Selected ion transitions with optimized collision energies (CE), collision cell exit potential (CXP), declustering potential (DP), quantifier A(1) to qualifier A(2) ratio and retention time (Rt) for each analyte, Table S4: Monoisotopic calculated exact masses (EM) and measured accurate masses (AM) of negatively loaded alternariol sulfate ion, alternariol mono methylether sulfate ion and altenuisol sulfate ion, Table S5: Limits of detection (LODs) and limits of quantification (LOQs) determined according to DIN EN ISO 32645.
