# Supplementary material for: Spotlight on the Underdogs—An Analysis of Underrepresented Alternaria Mycotoxins Formed Depending on Varying Substrate, Time and Temperature Conditions
Source: Toxins (Basel). 2016 Nov 19;8(11):344. doi: 10.3390/toxins8110344 (PMC5127140; doi:10.3390/toxins8110344)
Supplement: Supplementary file 1 [file toxins-08-00344-s001.docx]

Supplementary Materials: Spotlight on the Underdogs—An Analysis of Underrepresented *Alternaria* Mycotoxins Formed in Dependence on Varying Substrate, Time and Temperature Conditions

Theresa Zwickel *, Sandra M. Kahl, Horst Klaffke, Michael Rychlik and Marina E. H. Müller *

**Table S1.** Mean content (*n* = 3) of produced *Alternaria* toxins at 7 °C after 4, 7 and 14 days in wheat and in rice in mg·kg^−1^ (± standard error of the mean; n.d. not detected).

| **7 °C** | **4 d** | **4 d** | **7 d** | **7 d** | **14 d** | **14 d** |
| --- | --- | --- | --- | --- | --- | --- |
|  | **Wheat** | **Rice** | **Wheat** | **Rice** | **Wheat** | **Rice** |
|  | **Mean Content mg·kg^−1^ ± Standard Error of the Mean mg·kg^−1^** | | | | | |
| **GH15t** |  | | | | | |
| ATX-I | n.d. | n.d. | n.d. | n.d. | n.d. | n.d. |
| ATX-II | n.d. | n.d. | n.d. | n.d. | n.d. | n.d. |
| STTX-III | n.d. | n.d. | **0.244** ± 0.068 | n.d. | **13.8** ± 3.6 | **57.9** ± 17.4 |
| AOH | n.d. | n.d. | **0.0151** ± 0.0005 | n.d. | **0.023** ± 0.005 | **0.154** ± 0.052 |
| AME | n.d. | n.d. | n.d. | n.d. | n.d. | n.d. |
| Σ(iso)ALT | n.d. | n.d. | n.d. | n.d. | n.d. | n.d. |
| ATL | n.d. | n.d. | n.d. | n.d. | n.d. | n.d. |
| AA-III | **0.181** ± 0.029 | n.d. | **0.108** ± 0.004 | n.d. | **0.112** ± 0.004 | **0.217** ± 0.019 |
| TEN | n.d. | n.d. | n.d. | n.d. | n.d. | n.d. |
| TeA | n.d. | n.d. | **0.0232** ± 0.0031 | **0.0151**± 0.0043 | **0.956** ± 0.228 | **110** ± 60 |
| **RN01Ct** |  |  |  |  |  |  |
| ATX-I | n.d. | n.d. | n.d. | n.d. | n.d. | n.d. |
| ATX-II | n.d. | n.d. | n.d. | n.d. | n.d. | n.d. |
| STTX-III | n.d. | n.d. | **0.139** ± 0.084 | **0.0414** ± 0.0041 | **15.7** ± 2.0 | **4.61** ± 0.79 |
| AOH | **0.0108 *** | **0.0277 *** | **0.0733** ± 0.0436 | n.d. | **0.0448** ± 0.0155 | **0.0317** ± 0.0023 |
| AME | n.d. | n.d. | n.d. | n.d. | n.d. | n.d. |
| Σ(iso)ALT | n.d. | n.d. | n.d. | n.d. | n.d. | n.d. |
| ATL | n.d. | n.d. | n.d. | n.d. | n.d. | n.d. |
| AA-III | **0.0752** ± 0.0263 | n.d. | **0.121** ± 0.015 | **0.0640** ± 0.0100 | **0.0879** ± 0.0159 | **0.0903** ± 0.0167 |
| TEN | n.d. | n.d. | n.d. | n.d. | n.d. | n.d. |
| TeA | **0.0462** ± 0.0247 | **1.58** ± 1.20 | **1.79** ± 0.82 | **0.405** ± 0.033 | **139** ± 32 | **252** ± 66 |
| **RN04Ci** |  |  |  |  |  |  |
| ATX-I | n.d. | n.d. | n.d. | n.d. | n.d. | n.d. |
| ATX-II | n.d. | n.d. | n.d. | n.d. | n.d. | n.d. |
| STTX-III | n.d. | n.d. | **0.0624** ± 0.0191 | **0.403** ± 0.087 | **19.5** ± 2.7 | **163** ± 17 |
| AOH | n.d. | n.d. | n.d. | n.d. | **0.0217 *** | n.d. |
| AME | n.d. | n.d. | n.d. | n.d. | n.d. | n.d. |
| Σ(iso)ALT | n.d. | n.d. | n.d. | n.d. | n.d. | n.d. |
| ATL | n.d. | n.d. | n.d. | n.d. | n.d. | n.d. |
| AA-III | **0.0862** ± 0.0474 | n.d. | **0.0600** ± 0.0104 | **0.0719** ± 0.0080 | **0.142** ± 0.009 | **0.0998** ± 0.0022 |
| TEN | n.d. | n.d. | n.d. | n.d. | n.d. | n.d. |
| TeA | **0.195** ± 0.121 | n.d. | **0.0156** * | **0.0181** ± 0.0022 | n.d. | n.d. |

* Only one value above LOQ detected.

**Table S2.** Mean content (*n* = 3) of produced *Alternaria* toxins at 25 °C after 4, 7 and 14 days in wheat and in rice in mg·kg^−1^ (± standard error of the mean; n.d. not detected).

| **25 °C** | **4 d** | **4 d** | **7 d** | **7 d** | **14 d** | **14 d** |
| --- | --- | --- | --- | --- | --- | --- |
|  | **Wheat** | **Rice** | **Wheat** | **Rice** | **Wheat** | **Rice** |
|  | **Mean Content mg·kg^−1^ ± Standard Error of the Mean mg·kg^−1^** | | | | | |
| **GH15t** |  | | | | | |
| ATX-I | n.d. | **0.791** ± 0.070 | **0.545** ± 0.352 | **10.5** ± 2.8 | **20.4** ± 3.8 | **27.0** ± 5.5 |
| ATX-II | n.d. | **4.55** ± 2.32 | **4.07** ± 2.49 | **14.8** ± 7.0 | **36.2** ± 0.4 | **13.9** ± 3.5 |
| STTX-III | **14.9** ± 1.5 | **167** ± 79 | **122** ± 62 | **251** ± 98 | **402** ± 12 | **206** ± 34 |
| AOH | **0.311** ± 0.062 | **96.1** ± 40.0 | **22.4** ± 2.8 | **570 ^#^** (87.1; 62.5) | **74.1** ± 4.4 | **346 ^#^** (48.0; 40.4) |
| AME | **0.0776** ± 0.0334 | **20.4** ± 10.1 | **2.44** ± 1.32 | **674 ^#^** (37.4; 22.5) | **110** ± 13 | **278 ^#^** (25.2; 38.7) |
| Σ(iso)ALT | **0.0091 *** | **0.516** ± 0.244 | **1.54** ± 4.6 | **3.29** ± 1.29 | **31.7** ± 7.8 | **39.6** ± 9.5 |
| ATL | **0.0876** ± 0.0075 | **3.19** ± 2.0 | **1.11** ± 0.55 | **11.3** ± 5.4 | **12.8** ± 0.9 | **10.2** ± 3.6 |
| AA-III | **0.170** ± 0.048 | **0.543** ± 0.250 | **33.0** ± 12.8 | **7.29** ± 4.30 | **102** ± 10 | **12.9** ± 3.1 |
| TEN | **21.3** ± 2.9 | **32.4** ± 7.7 | **91.6** ± 6.1 | **82.2** ± 19.9 | **185** ± 12 | **144** ± 15 |
| TeA | **2053** ± 48 | **3358** ± 308 | **4309** ± 282 | **2946** ± 267 | **3706** ± 133 | **3327** ± 367 |
| **RN01Ct** |  |  |  |  |  |  |
| ATX-I | n.d. | **0.282** ± 0.111 | **0.732** ± 0.244 | **4.39** ± 1.83 | **29.5** ± 10.0 | **79.7** ± 16.4 |
| ATX-II | **0.489** ± 0.076 | **8.05** ± 2.58 | **19.3** ± 8.8 | **28.9** ± 12.3 | **34.5** ± 3.1 | **46.1** ± 7.0 |
| STTX-III | **38.7** ± 0.8 | **225** ± 47 | **380** ± 185 | **470** ± 244 | **383** ± 22 | **604** ± 96 |
| AOH | **13.8** ± 3.1 | **39.7** ± 7.1 | **1030** ^#^(205; 281) | **6452** ^#^(9.05; 514) | **2289** ± 159 | **9693** ± 144 |
| AME | **0.712** ± 0.102 | **5.98** ± 2.13 | **506** ^#^(33; 36) | **2782** ^#^(0.82; 180) | **717** ± 7 | **831** ± 20 |
| Σ(iso)ALT | **0.169** ± 0.046 | **0.249** ± 0.048 | **31.6** ± 6.8 | **19.3 *** | **438** ± 68 | **280** ± 37 |
| ATL | **0.401** ± 0.055 | **1.24** ± 0.37 | **15.9** ± 5.5 | **21.0** ± 12.7 | **41.2** ± 2.7 | **84.2** ± 8.8 |
| AA-III | **7.67** ± 1.09 | **1.20** ± 0.60 | **163** ± 20 | **29.0 *** | **375** ± 27 | **84** ± 22 |
| TEN | **3.86** ± 0.26 | **9.06** ± 0.30 | **17.6** ± 1.7 | **7.09** ± 3.50 | **54.9** ± 13.0 | **22.4** ± 2.6 |
| TeA | **1984** ± 188 | **4801** ± 144 | **6098** ± 437 | **4637** ± 342 | **5995** ± 447 | **5464** ± 749 |
| **RN04Ci** |  |  |  |  |  |  |
| ATX-I | n.d. | **0.472** ± 0.247 | n.d. | **1.27** ± 0.03 | n.d. | **1.04** ± 0.05 |
| ATX-II | n.d. | n.d. | n.d. | **0.219** ± 0.017 | n.d. | **0.173 *** |
| STTX-III | n.d. | **315** ± 144 | **0.501** ± 0.333 | **563** ± 99 | **1.51** ± 0.205 | **277** ± 64 |
| AOH | n.d. | **0.0249** ± 0.0016 | n.d. | **0.153** ± 0.067 | **0.0212** ± 0.0024 | **0.391** ± 0.073 |
| AME | n.d. | n.d. | n.d. | **0.0298 *** | **0.0318** ± 0.0091 | **0.139** ± 0.017 |
| Σ(iso)ALT | n.d. | **0.0135** ± 0.0011 | n.d. | **0.0603 *** | n.d. | **0.0398** ± 0.0013 |
| ATL | n.d. | n.d. | **0.144** ± 0.004 | **0.106 *** | **0.348** ± 0.017 | **0.618** ± 0.043 |
| AA-III | **0.104** ± 0.009 | **0.214** ± 0.019 | **0.144** ± 0.017 | **0.515** ± 0.097 | **0.167** ± 0.001 | **0.395** ± 0.066 |
| TEN | n.d. | n.d. | n.d. | n.d. | n.d. | **0.729 *** |
| TeA | **0.278 *** | **1.79** ± 0.05 | **0.581** ± 0.294 | **1.86** ± 0.56 | **0.780** ± 0.359 | **1.59** ± 0.37 |

* Only one value above LOQ detected; ^#^ Strongly varying values (eliminated values in brackets); highest produced values were chosen.

**Table S3.** Selected ion transitions with optimized collision energies (CE), collision cell exit potential (CXP), declustering potential (DP), quantifier A(1) to qualifier A(2) ratio and retention time (R_t_) for each analyte.

| **Compound Name** | **Q1 Mass  (Da)** | **Q3 Mass  (Da)** | **DP  (Volt)** | **CE  (Volt)** | **CXP  (Volt)** | **A(1)/A(2)  ION Ratio** | **R_t_ (min)** |
| --- | --- | --- | --- | --- | --- | --- | --- |
| **AAL TB1 + TB2 ^1^** | 504.3 | 156.8 | −80 | −34 | −11 | 5.1 | 3.98 |
| **AAL TB1 + TB2 ^2^** | 504.3 | 112.9 | −80 | −52 | −9 |  | 3.98 |
| **ALT ^1^** | 291.0 | 229.1 | −50 | −20 | −13 | 1.8 | 4.21 |
| **ALT ^2^** | 291.0 | 246.9 | −50 | −28 | −11 |  | 4.21 |
| **AA-III ^1^** | 320.9 | 233.1 | −70 | −22 | −13 | 0.9 | 3.72 |
| **AA-III ^2^** | 320.9 | 277.0 | −70 | −18 | −15 |  | 3.72 |
| **ATL ^1^** | 272.9 | 258.1 | −90 | −32 | −15 | 5.3 | 3.84 |
| **ATL ^2^** | 272.9 | 174.1 | −90 | −44 | −15 |  | 3.84 |
| **AOH ^1^** | 257.0 | 214.9 | −80 | −36 | −15 | 1.8 | 3.98 |
| **AOH ^2^** | 257.0 | 147.1 | −80 | −46 | −9 |  | 3.98 |
| **AME ^1^** | 270.9 | 256.1 | −75 | −30 | −11 | 4.2 | 4.62 |
| **AME ^2^** | 270.9 | 228.0 | −75 | −40 | −17 |  | 4.62 |
| **ATX I ^1^** | 351.0 | 333.1 | −70 | −14 | −11 | 1.0 | 4.33 |
| **ATX I ^2^** | 351.0 | 315.0 | −70 | −22 | −9 |  | 4.33 |
| **ATX-II ^1^** | 349.0 | 330.9 | −85 | −18 | −17 | 4.4 | 4.48 |
| **ATX-II ^2^** | 349.0 | 313.0 | −85 | −30 | −17 |  | 4.48 |
| **isoALT ^1^** | 291.0 | 246.9 | −65 | −18 | −13 | 5.2 | 4.21 |
| **isoALT ^2^** | 291.0 | 229.1 | −65 | −40 | −13 |  | 4.21 |
| **STTX-III ^1^** | 347.0 | 300.9 | −85 | −30 | −17 | 0.9 | 4.51 |
| **STTX-III ^2^** | 347.0 | 328.9 | −85 | −22 | −15 |  | 4.51 |
| **TEN ^1^** | 413.0 | 141.0 | −85 | −28 | −7 | 1.6 | 4.37 |
| **TEN ^2^** | 413.0 | 270.9 | −85 | −22 | −11 |  | 4.37 |
| **TeA ^1^** | 196.0 | 138.9 | −50 | −26 | −9 | 1.1 | 3.78 |
| **TeA ^2^** | 196.0 | 111.9 | −50 | −32 | −7 |  | 3.78 |
| **AME-Sulf ^1^** | 351.1 | 271 | −50 | −28 | −11 | 2.7 | 4.40 |
| **AME-Sulf ^2^** | 351.1 | 256 | −50 | −40 | −17 |  | 4.40 |
| **AOH-Sulf ^1^** | 337.1 | 257 | −50 | −30 | −15 | 3.8 | 3.70 |
| **AOH-Sulf ^2^** | 337.1 | 213 | −50 | −46 | −9 |  | 3.70 |
| **ATL-Sulf ^1^** | 353.1 | 273 | −50 | −30 | −15 | 2.9 | 3.65 |
| **ATL-Sulf ^2^** | 353.1 | 230 | −50 | −44 | −15 |  | 3.65 |

^1^ MRM used as the quantifier transition; ^2^ MRM used as a qualifier transition; * Calculated as sum of ALT and isoALT (Σ(iso)ALT).

**Table S4.** Monoisotopic calculated exact masses (EM) and measured accurate masses (AM) of negatively loaded alternariol sulfate ion, alternariol mono methylether sulfate ion and altenuisol sulfate ion.

|  |  | **EM (Da)** | **AM (Da)** |
| --- | --- | --- | --- |
| AOH-sulfate ion | [C_14_H_9_O_8_S]^−^ | 337.0024 | 337.0029 |
| AME-sulfate ion | [C_15_H_11_O_8_S]^−^ | 351.0180 | 351.0186 |
| ATL-sulfate ion | [C_14_H_9_O_9_S]^−^ | 352.9973 | 352.9977 |

**Table S5.** Limits of detection **(**LODs) and limits of quantification (LOQs) determined according to DIN EN ISO 32645.

| **Analyte** | **Range  [μg·L^−1^]** | **R^2^** | **LOD  [μg·kg^−1^]** | **LOQ  [μg·kg^−1^]** |
| --- | --- | --- | --- | --- |
| **ALT** | 0.25–4.5 | 0.963 | 3.0 | 9.5 |
| **AME** | 0.25–4.5 | 0.999 | 0.5 | 1.8 |
| **AOH** | 0.50–4.5 | 0.998 | 0.6 | 2.1 |
| **TEA** | 0.25–4.5 | 0.990 | 1.4 | 4.6 |
| **TEN** | 0.25–4.5 | 0.996 | 0.9 | 3.1 |
| **AAL TB** | 0.25–4.5 | 0.996 | 1.7 | 5.5 |
| **ATX-I** | 0.50–4.5 | 0.994 | 1.1 | 3.7 |
| **ATX-II** | 0.50–4.5 | 0.995 | 1.0 | 3.4 |
| **STTX-III** | 0.25–4.5 | 0.996 | 0.9 | 3.2 |
| **AA-III** | 0.25–4.5 | 0.996 | 0.9 | 3.1 |
| **ATL** | 0.50–4.5 | 0.994 | 1.2 | 3.7 |
| **Parameters** | |  |  | |
| ***N*** | | 10 | number of calibration points | |
| **max. permissible uncertainty** | | 33.33 | in % | |
| ***k*** | | 3.000 | k-Factor for LOQ calculation | |
| ***t*_one-sided_** | | 1.860 | one-sided, N-2, 95% | |
| ***t*_two-sided_** | | 2.306 | two-sided, N-2, 95% | |
| **Significance level_1_** | | 0.05 | confidence interval and *t*-Test | |
| **Significance level_2_** | | 0.01 | Mandel fitting test | |
